# Dynamic Functional Connectivity Predicts Treatment Response to Electroconvulsive Therapy in Major Depressive Disorder

**DOI:** 10.3389/fnhum.2021.689488

**Published:** 2021-07-06

**Authors:** Hossein Dini, Mohammad S. E. Sendi, Jing Sui, Zening Fu, Randall Espinoza, Katherine L. Narr, Shile Qi, Christopher C. Abbott, Sanne J. H. van Rooij, Patricio Riva-Posse, Luis Emilio Bruni, Helen S. Mayberg, Vince D. Calhoun

**Affiliations:** ^1^Department of Architecture, Design and Media Technology, Aalborg University, Copenhagen, Denmark; ^2^Wallace H. Coulter Department of Biomedical Engineering at Georgia Institute of Technology and Emory University, Atlanta, GA, United States; ^3^Department of Electrical and Computer Engineering at Georgia Institute of Technology, Atlanta, GA, United States; ^4^Tri-Institutional Center for Translational Research in Neuroimaging and Data Science, Georgia Institute of Technology, Georgia State University, Emory University, Atlanta, GA, United States; ^5^National Laboratory of Pattern Recognition, Institute of Automation, Chinese Academy of Sciences, Beijing, China; ^6^Departments of Neurology, Psychiatry and Biobehavioral Sciences, University of California, Los Angeles, Los Angeles, CA, United States; ^7^Department of Psychiatry, University of New Mexico, Albuquerque, NM, United States; ^8^Department of Psychiatry and Behavioral Sciences, Emory University School of Medicine, Atlanta, GA, United States; ^9^Departments of Neurology, Neurosurgery, Psychiatry and Neuroscience, Center for Advanced Circuit Therapeutics, Icahn School of Medicine at Mount Sinai, New York, NY, United States

**Keywords:** major depressive disorder, electroconvulsive therapy, dynamic functional network connectivity, resting-state functional MRI, prediction, default mode network, cognitive control network

## Abstract

**Background:** Electroconvulsive therapy (ECT) is one of the most effective treatments for major depressive disorder. Recently, there has been increasing attention to evaluate the effect of ECT on resting-state functional magnetic resonance imaging (rs-fMRI). This study aims to compare rs-fMRI of depressive disorder (DEP) patients with healthy participants, investigate whether pre-ECT dynamic functional network connectivity network (dFNC) estimated from patients rs-fMRI is associated with an eventual ECT outcome, and explore the effect of ECT on brain network states.

**Method:** Resting-state functional magnetic resonance imaging (fMRI) data were collected from 119 patients with depression or depressive disorder (DEP) (76 females), and 61 healthy (HC) participants (34 females), with an age mean of 52.25 (*N* = 180) years old. The pre-ECT and post-ECT Hamilton Depression Rating Scale (HDRS) were 25.59 ± 6.14 and 11.48 ± 9.07, respectively. Twenty-four independent components from default mode (DMN) and cognitive control network (CCN) were extracted, using group-independent component analysis from pre-ECT and post-ECT rs-fMRI. Then, the sliding window approach was used to estimate the pre-and post-ECT dFNC of each subject. Next, k-means clustering was separately applied to pre-ECT dFNC and post-ECT dFNC to assess three distinct states from each participant. We calculated the amount of time each subject spends in each state, which is called “occupancy rate” or OCR. Next, we compared OCR values between HC and DEP participants. We also calculated the partial correlation between pre-ECT OCRs and HDRS change while controlling for age, gender, and site. Finally, we evaluated the effectiveness of ECT by comparing pre- and post-ECT OCR of DEP and HC participants.

**Results:** The main findings include (1) depressive disorder (DEP) patients had significantly lower OCR values than the HC group in state 2, where connectivity between cognitive control network (CCN) and default mode network (DMN) was relatively higher than other states (corrected *p* = 0.015), (2) Pre-ECT OCR of state, with more negative connectivity between CCN and DMN components, is linked with the HDRS changes (R = 0.23 corrected *p* = 0.03). This means that those DEP patients who spent less time in this state showed more HDRS change, and (3) The post-ECT OCR analysis suggested that ECT increased the amount of time DEP patients spent in state 2 (corrected *p* = 0.03).

**Conclusion:** Our finding suggests that dynamic functional network connectivity (dFNC) features, estimated from CCN and DMN, show promise as a predictive biomarker of the ECT outcome of DEP patients. Also, this study identifies a possible underlying mechanism associated with the ECT effect on DEP patients.

## Introduction

Major depressive disorder is a debilitating brain disorder (Tsuchiyagaito et al., 2021), which is characterized by impaired cognitive functioning, such as inattention and inability to focus, somatic abnormalities, and neurovegetative symptoms, such as sleep and appetite disturbance (Liu et al., 2021; Luo et al., 2021). Based on the global burden of a disease report from the World Health Organization, depression is the third rank cause of disability and has been estimated to be the first-rank cause of burden before 2030 (World Health Organization, 2008; Ebneabbasi et al., 2021). There are effective treatments accessible such as psychotherapy and chemical antidepressants, but about 30 percent of patients suffering from major depressive disorder (MDD) do not respond to these treatments (Rush et al., 2006). Therefore, there is an essential need for advanced therapies, such as deep brain stimulation (DBS), transcranial magnetic stimulation (TMS), and electroconvulsive therapy (ECT), which are indicated for treatment resistant depression (Settell et al., 2017; Mo et al., 2020; Williams et al., 2021).

Among all mentioned therapies, ECT can be considered as one of the most effective treatments for pharmacological resistant MDD (Enneking et al., 2020) due to its faster action and higher remission rate than typical medicine-based treatments (UK ECT Review Group, 2003). One hundred thousand annual ECT treatments in the U.S revealed that the success rate of this treatment is around 75%, with typical remission rates within 3–4 weeks (Hermann et al., 1995; Weiner and American Psychiatric Association, 2001). Moreover, pretreatment clinical and demographic characteristics are poorly associated with eventual treatment response (Haq et al., 2015). Therefore, understanding the underlying neural and cognitive mechanisms behind the mechanism of action of ECT, potentially, could increase the efficacy of treatment, and pretreatment biomarkers that are associated with eventual response are needed.

In recent years, functional network connectivity (FNC) data obtained from resting-state functional magnetic resonance imaging (rs-fMRI) time series has demonstrated highly informative about the underlying brain connectivity patterns in mental disorders, such as MDD (Mulders et al., 2015; Yan et al., 2019; Liu et al., 2020; Luo et al., 2021). Recently, studies have shown that ECT resets and stimulates the formation of the brain regions/networks connectivity (Wang H. et al., 2018; Wang J. et al., 2018; Bai et al., 2020). Investigations of the whole-brain FNC of the patients with depression showed a reduction in the left dorsal lateral prefrontal cortex connectivity corresponded to ECT therapeutic course (Perrin et al., 2012; Abbott et al., 2013). Several recent studies have reported functional and structural connectivity changes occurred in the amygdala and anterior cingulate cortex (ACC) after ECT (Wang et al., 2017; Takamiya et al., 2018; Gryglewski et al., 2019; Qiu et al., 2019; Sartorius et al., 2019). Another study declared that the cognitive control network (CCN) and the default mode network (DMN) play a vital role as the most effective brain network in regulating brain connections after ECT (Menon, 2015). Moreover, ECT-mediated connectivity changes include increased intra-network connectivity in CCN (Wang H. et al., 2018; Wang J. et al., 2018), decreased dorsolateral prefrontal cortex global functional connectivity (DLPFC) as a part of CCN (Perrin et al., 2012), and connectivity changes in the DMN (Mulders et al., 2016; Wei et al., 2018; Bai et al., 2019).

In the aforementioned studies, FNC estimated from CCN and DMN is often assumed to be static over time. However, this assumption runs contrary to the dynamic nature of brain FNC. Dynamic FNC (dFNC) has been recently introduced to overcome this limitation (Allen et al., 2014; Zendehrouh et al., 2020; Sendi et al., 2021c). Dynamic FNC refers to brain connectivity within subintervals of the time series, as opposed to static FNC, which reflects averaged brain connections over an entire scan (Calhoun et al., 2014). In recent years, dFNC estimated from rs_fMRI time series has been highly informative about the underlying different brain regions connectivity patterns in various brain disorders, including schizophrenia, MDD, and Alzheimer's disease (Sendi et al., 2020, 2021a,c). As a result, we hypothesized that looking at the effect of ECT on dFNC might reveal how and to what extent ECT affects dynamic connectivity changes in the DMN and CCN.

In this study, we used rs-fMRI data from 119 patients with depression (DEP), who experienced a series of ECT and 61 HC controls to find the neural mechanisms behind the improvement after ECT and find associations with the effectiveness of ECT before applying it. To this aim, we used group independent component analysis (ICA) and extracted independent components from DMN and CCN and estimated dFNC in these two networks by applying a sliding window approach and clustered dFNC into a few brain states, using k-means clustering. Finally, we compared the occupancy rate (OCR) estimated from a state vector, an output of k-means clustering, between HC and DEP in both pre- and post-ECT. Moreover, correlating OCR with clinical data, we assessed pre-ECT rs-fMRI dFNC patterns associated with post-ECT depression outcomes.

## Materials and Methods

### Participants and Clinical Outcome

This study used neuroimaging, clinical, and demographic information of 119 patients (76 females) diagnosed with depression (called “DEP” hereafter) and 61 healthy (HC) subjects (34 females) from either University of New Mexico (UNM) or the University of California Los Angeles (UCLA). Exclusion criteria were as follows: (1) Having any neurodegenerative and neurological disorders, such as Alzheimer's disease or psychiatric conditions, such as schizophrenia; (2) Having alcohol or drug addiction, pregnancy; and (3) potential dangers under magnetic resonance imaging (MRI), such as using a pacemaker.

Hamilton Depression Rating Scale-17 items (HDRS) were used to assess the symptom severity of the patient group before and after the ECT (Heijnen et al., 2010). Initial and final assessments were given to the participants before ECT started and within a week of completing ECT series, respectively. UNM subjects received concurrent psychotropic, but UCLA subjects discontinued psychotropic medications before the ECT outset. Demographical information and clinical measurements can be seen in Table 1. Finally, all the participants signed the consent form, and this study has been approved by the institutional review boards at UNM and UCLA.

**Table 1:** Demographic and clinical details of the participants for each site.

		**DEP**	**HC**	***P*-value**
UCLA	Number	45	33	NA
	Age	41.22 ± 13.51	39.03 ± 12.21	0.46
	Gender(M/F)	20/25	15/18	0.99
	Pre-ECT HDRS	25.17 ± 6.15	NA	NA
	Post-ECT HDRS	16.22 ± 9.33	NA	NA
	Number of treatments	8.84 ± 3.40	NA	NA
	Antidepressants (%)	0/45 (0.0)	NA	NA
UNM	Number	74	28	NA
	Age	64.99 ± 9.09	60.22 ± 8.02	0.02
	Gender(M/F)	23/51	11/16	0.62
	Pre-ECT HDRS	25.85 ± 6.13	NA	NA
	Post-ECT HDRS	16.90 ± 6.70	NA	NA
	Number of treatments	8.92 ± 2.86	NA	NA
	Antidepressants (%)	14/74 (0.18)	NA	NA
Total	Number	119	61	NA
	Age	55.94 ± 15.87	48.56 ± 14.90	0.008
	Gender(M/F)	43/76	26/34	0.99
	Pre-ECT HDRS	25.59 ± 6.14	NA	NA
	Post-ECT HDRS	11.48 ± 9.07	NA	NA
	Number of treatments	8.89 ± 3.07	NA	NA
	Antidepressants(%)	14/119 (0.11)	NA	NA

*M, Male; F, Female; ECT, Electroconvulsive therapy; HDRS, Hamilton depression rating scale; DEP, Depression; HC, Healthy control*.

### ECT Procedure

In the UNM site, Thymatron System IV (Somatics, Lake Bluff, IL, USA) was used, and the ECT procedure was initiated with a right unilateral d'Elia (ultra-brief pulse width of 0.3 ms, a stimulus dosage at 6 × threshold) placement of electrodes. All the participants started the first ECT session with the right unilateral electrode placement. The ECT non-response subjects, then, received bitemporal electrode placement (brief pulse width of 1 ms (UNM) or .5 ms (UCLA), a stimulus dosage at 2 × threshold). At both sites, a Mecta 5000Q (MECTA Corp., Tualatin, OR, USA) was used for ECT administration. Treatments were applied three times a week until obtaining a stable clinical response or psychiatrist decision to stop treatment in the context of nonresponse. ECT implementation procedure followed the clinical standards announced by the APA ECT Task Force Report and was not manipulated for the goal of this study. During the treatment process, the patients were oxygenated and received adequate induction (methohexital or etomidate) and relaxation (succinylcholine). Clinical measures such as blood pressure were monitored during the treatment.

### fMRI Data Acquisition

At the UNM site, a 3-T Siemens Trio scanner (Siemens Healthcare, Malvern, PA, USA) was used to collect MRI data. Parameters of the whole-brain gradient-echo echo-planar imaging sequence are as follows: echo time (TE) = 29 milliseconds (ms), repetition time (TR) = 2 s (s), voxel size = 3.75 × 3.75 × 4.55 mm, ip angle (FA), 75°, and 154 volumes. At UCLA, a 3-Tesla MAGNETOM Allegra MRI scanner (Siemens, Erlangen, Germany) was used to collect MRI data. Parameters of functional images are as follows: TE = 30 ms, TR = 2 s, voxel size = 3.4 × 3.4 × 5 mm, FA = 70°, and 180 volumes. The duration of resting-state scans was a minimum of 5 min and 16 s, and the participants were guided to passively keep their concentration on the fixation cross during the scan.

### Data Pre-processing

The standard preprocessing steps for fMRI data, using statistical parametric mapping (SPM12, https://www.fil.ion.ucl.ac.uk/spm/), include the following: (1) to address longitudinal relaxation effects, five initial fMRI scans were removed; (2) time differences in slice acquisition were corrected; (3) motion was corrected, using SPM; (4) imaging data were spatially normalized based on an echo-planar imaging (EPI) template in standard Montreal Neurological Institute (MNI) space and was resampled to 3 × 3 × 3 mm^3^; and (5) a 6-mm full-width half-maximum (FWHM) Gaussian kernel for spatial smoothing is applied to the data.

The Neuromark fully automated group ICA pipeline, using GIFT (http://trendscenter.org/software/gift), is implemented to extract reliable CCN and DMN-independent components (ICs). In this method, previously derived components maps were used as priors for spatially constrained ICA. Considering group-level spatial maps from two large-sample HC datasets, replicable components were identified and used for the spatial priors (Du et al., 2020). Prior to the dynamic functional connectivity method, we implemented de-noising and artifact rejection steps as follows: (1) linear, quadratic, and cubic de-trending; (2) use of six realignment parameters and their temporal derivatives for multiple regression; and 3) outlier removal and band-pass filtering (from 0.01 to 0.15 Hz). Identified components in the cognitive control network (CCN) and default mode network (DMN) can be seen in Table 2.

**Table 2:** Component labels.

		**Component name**	**Peak coordinate (mm)**		
1	CCN	Inferior parietal lobule ([IPL], 68)	45.5	−61.5	43.5
2		Insula (33)	−30.5	22.5	−3.5
3		Superior medial frontal gyrus ([SMFG], 43)	−0.5	50.5	29.5
4		Inferior frontal gyrus ([IFG], 70)	−48.5	34.5	−0.5
5		Right inferior frontal gyrus ([R IFG], 61)	53.5	22.5	13.5
6		Middle frontal gyrus ([MiFG], 55)	−41.5	19.5	26.5
7		Inferior parietal lobule ([IPL], 63)	−53.5	−49.5	43.5
8		Left inferior parietal lobue ([R IPL], 79)	44.5	−34.5	46.5
9		Supplementary motor area ([SMA], 84)	−6.5	13.5	64.5
10		Superior frontal gyrus ([SFG], 96)	−24.5	26.5	49.5
11		Middle frontal gyrus ([MiFG], 88)	30.5	41.5	28.5
12		Hippocampus ([HiPP], 48)	23.5	−9.5	−16.5
13		Left inferior parietal lobue ([L IPL], 81)	45.5	−61.5	43.5
14		Middle cingulate cortex ([MCC], 37)	−15.5	20.5	37.5
15		Inferior frontal gyrus ([IFG], 67)	39.5	44.5	−0.5
16		Middle frontal gyrus ([MiFG], 38)	−26.5	47.5	5.5
17		Hippocampus ([HiPP], 83)	−24.5	−36.5	1.5
18	DMN	Precuneus (32)	−8.5	−66.5	35.5
19		Precuneus (40)	−12.5	−54.5	14.5
20		Anterior cingulate cortex ([ACC], 23)	−2.5	35.5	2.5
21		Posterior cingulate cortex ([PCC], 71)	−5.5	−28.5	26.5
22		Anterior cingulate cortex ([ACC], 17)	−9.5	46.5	−10.5
23		Precuneus (51)	−0.5	−48.5	49.5
24		Posterior cingulate cortex ([PCC], 94)	−2.5	54.5	31.5

### Functional Network Connectivity

**Figure 1:**
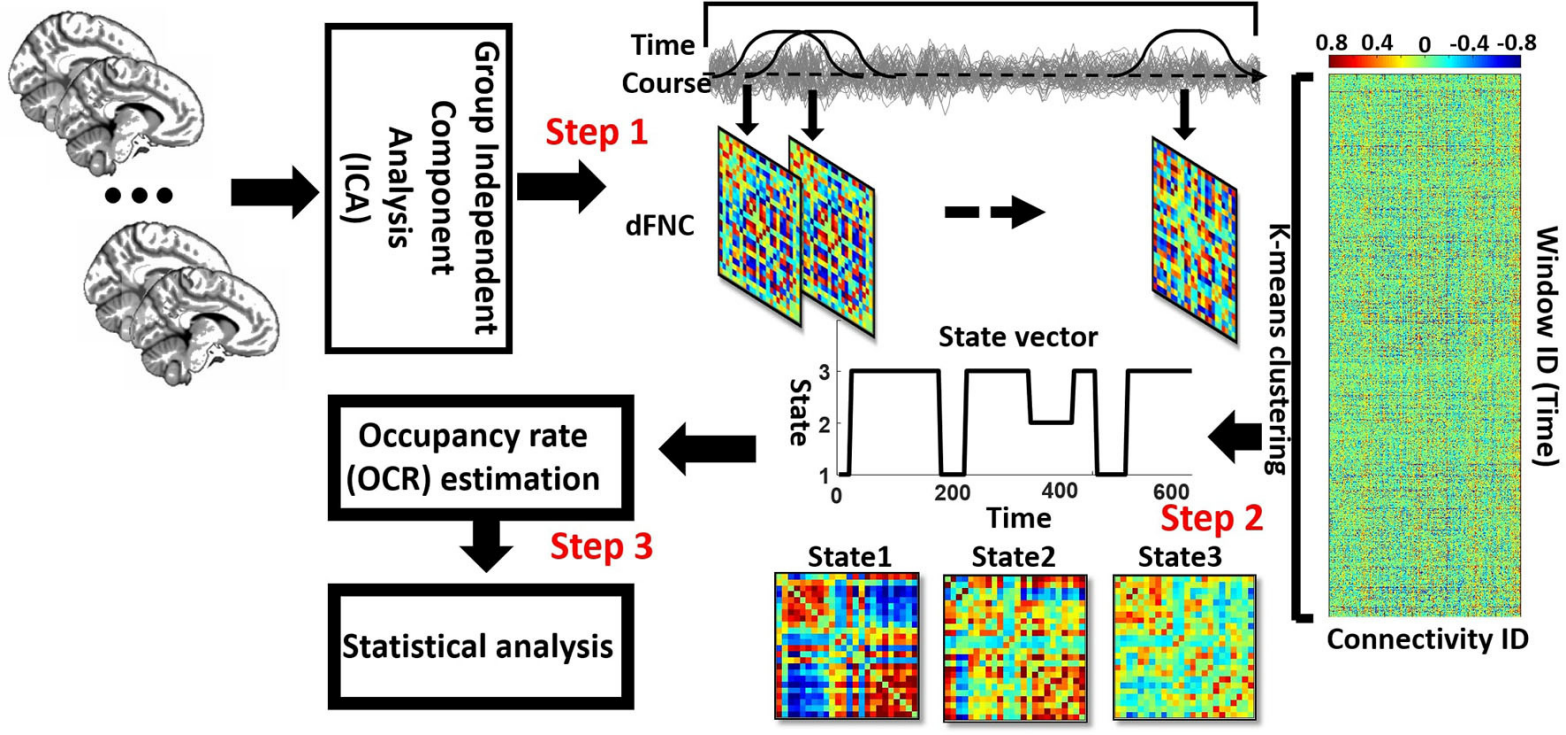
**Analytic pipeline:** The time-course signal of 24 components of the CCN and DMN networks has been identified, using group-independent component analysis (ICA). In step 2, a taper sliding window was used to segment the time-course signals and then calculated the functional network connectivity (FNC). After vectorizing the FNC matrixes, we have concatenated them, and then k-means clustering, *k* = 3, was used to group FNCs into three distinct states (Step 2). Elbow criteria were used to find the optimal k. In addition, the L1 distance metric is used in this clustering. Then, based on the state vector of each subject, the occupancy rate or OCR features-in total, three features-were calculated from the state vector of each subject. Then we compared the OCR among the groups by using a ks-test. Then, we adjusted all p-values by the Benjamini-Hochberg false discovery rate (FDR) correction in each analysis (Step 3).

A sliding window is a convolution of a rectangle (window size = 20 TRs = 40 s) with a Gaussian (σ = 3 s). This method was used to localize the dataset per time point, and the procedure can be seen in Figure 1, Step 1. Next, we used the Pearson correlation method to calculate dFNC between 24 sub-nodes of DMN and CCN. Then, we obtained 276 connectivity features (Figure 1, Step 1). Calculated dFNC for each window was concatenated for each subject as a form of C × C × T array (where C is the number of ICs and equals 276, and T represents total windows and equals 610). Finally, all arrays for all subjects were concatenated to show brain connectivity changes between ICs as a function of time (Figure 1, Step 2) (Allen et al., 2014; Sendi et al., 2021a,b).

### Clustering and dFNC Latent Features

We implemented a K-means clustering method on the output of the previous step, which is a concatenated dFNC between 24 ICs for all subjects, to separate the data into different clusters (Allen et al., 2014; Sendi et al., 2021c). We used the elbow criterion to calculate the optimum number of clusters (optimum k in the k-means method), a clustering analysis standard (Sendi et al., 2021c). This method defines the optimization equation as the distance of within-cluster and between clusters as a ratio and tries to minimize this ratio. We found the optimal number of clusters is 3, searching from k = 2 to 8. We used the L1 norm as our distance metric with 1,000 iterations. This process yielded three distinct states for the group of the participants and the state vector for each individual. The state vector shows the state of each brain and any given time. Subsequently, based on the state vector, we calculated the time interval of each subject (the number of time windows that each participant was in a specific state), and we call this feature the occupancy rate (OCR) of each state (Figure 1, Step 3). Thus, considering three states, we have three OCRs for each individual. Finally, we calculated the traveled distance for each subject, using Euclidean distance. To determine the traveled distance, we calculated the distance between any subsequent window of dFNC matrix and then summed up distance of all possible window pairs. Each subject has one traveled distance, which is a state-independent metric.

### Statistical Analysis

The occupancy rate (OCR) feature and the traveled distance between DEP and HC group are compared, using two sample Kolmogorov–Smirnov (ks-test), as a non-parametric test, because the distribution of estimated OCR was not normally distributed. This comparison was made on three features of OCRs and one traveled distance. Moreover, to see whether there is a link between pretreatment dFNC and the behavioral outcome, partial correlations assessed pre-ECT OCR and Hamilton Depression Rating Scale (HDRS) change accounting for age, gender, number of treatments, and a scanning site. All *p*-values were adjusted by the Benjamini-Hochberg method for a false discovery rate or FDR (Benjamini and Hochberg, 1995).

## Results

This section discusses the results obtained from dFNC analysis and the comparison between DEP and HC groups. It consists of dFNC states, resulting from clustering analysis, the correlation of OCR with HDRS scores, comparison between DEP and HC in OCR both in pre-ECT and post-ECT, and the traveled distance between the two groups.

### Clinical Results

The clinical and demographical information of the participants is provided in Table 1 separately for DEP and HC groups. The DEP pre-ECT HDRS was 25.6 (±6.1), and the post-ECT HDRS score was 11.5 (±9.1). Using a two-sample ks-test, we found a significant difference (*p* < 0.001) between HDRS values of pre- and post-ECT values.

### Dynamic Functional Network Connectivity States for Pre-ECT and Post-ECT

**Figure 2:**
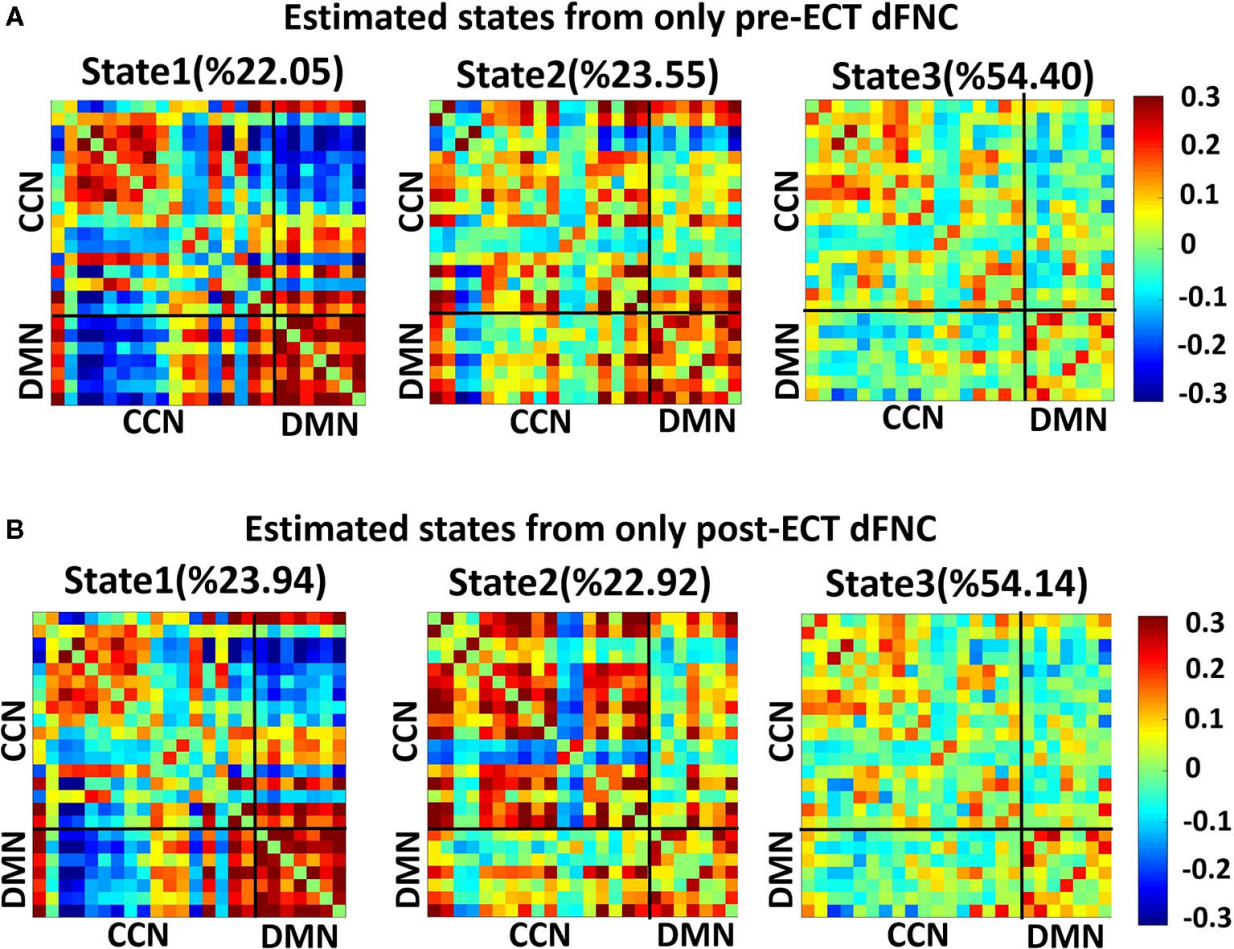
Dynamic functional connectivities in three identified states, using the clustering method, the input of clustering is combined with dFNC of all individuals (both HCs and DEPs). Each state consists of a 24 × 24 matrix where the positive connectivities are shown by hot color and negative connectivities are shown with cold colors. The values in parentheses show the overall percentage of time the participants spent in each specific state. **(A)** States resulted from clustering analysis of pre-ECT dFNC. **(B)** States resulted from clustering analysis of post-ECT dFNC.

We found three separate clusters (states), applying the k-means clustering method to dFNC of all subjects (both DEP and HC groups). We applied the clustering method to pre-ECT and post-ECT dFNC data separately. Figures 2A,B show these three distinct states pre-ECT and post-ECT, respectively. We found pre-ECT and post-ECT rs-fMRI generate similar brain states. To assess this similarity across corresponding states, we used Fisher correlation coefficients (state 1: R = 98.47%; state 2: R = 87.5%; state 3: R = 97.67%). Additionally, we calculated the average of dFNC values of CCN, DMN, and CCN/DMN (i.e., the connectivity between DMN and CCN) as shown in Table 3.

**Table 3:** The state-specific dFNC average.

		**CCN**	**DMN**	**CCN/DMN**
PRE-ECT	State 1	0.02 ± 0.16	0.27 ± 0.09	−0.03 ± 0.19
	State 2	0.05 ± 0.13	0.17 ± 0.08	0.05 ± 0.12
	State 3	0.04 ± 0.08	0.08 ± 0.08	−0.01 ± 0.07
Post-ECT	State 1	0.02 ± 0.15	0.28 ± 0.08	−0.01 ± 0.19
	State 2	0.10 ± 0.16	0.14 ± 0.09	0.04 ± 0.11
	State 3	0.03 ± 0.08	0.09 ± 0.08	−0.00 ± 0.07

*ECT, Electroconvulsive therapy; DMN, Default mode network; CCN, Cognitive control network*.

We found that both state 2 and state 3 have higher within-CCN connectivity than state 1 in pre-ECT (states 1– 2: corrected *p* = 0.02, and states 1–3: corrected *p* = 0.04). While state 1 had more increased within-DMN connectivity than the other two states in both pre-ECT and post-ECT (in pre-ECT: corrected p (states 1–2) <0.00, and corrected p (states 1–3) <0.00. In post-ECT: corrected *p* (states 1–2) <0.00 and corrected p (states 1–3) <0.00), only state 2 showed positive functional connectivity between DMN and CCN in both pre-ECT and post-ECT data (all the corrected p-values for all combinations are <0.00).

### Comparison of OCR and Traveled Distance Between HC and DEP in Pre-ECT and Post-ECT

**Figure 3:**
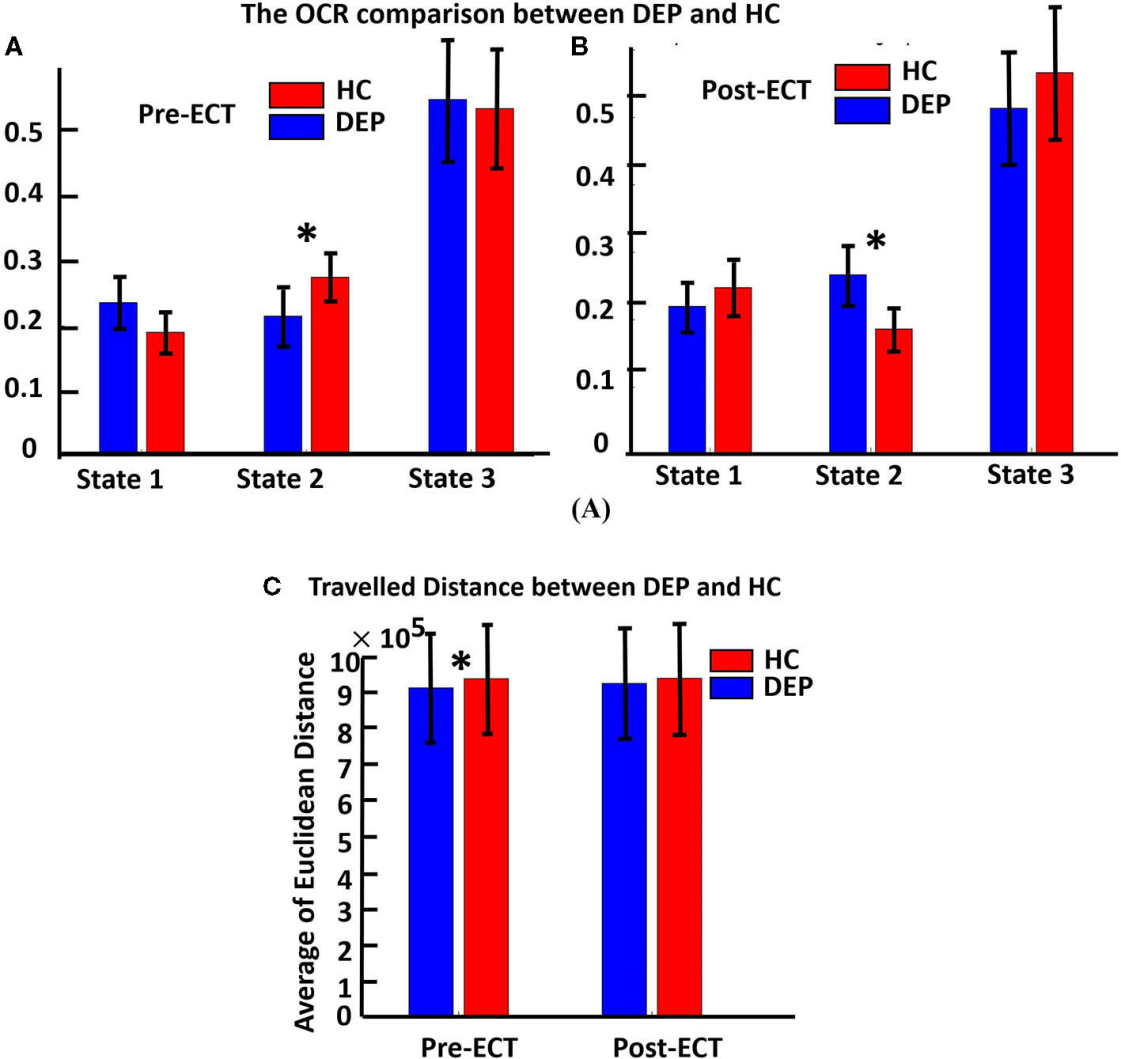
**(A)** The OCR comparison between DEP and HC in three distinct states of pre-ECT. Red bars indicating the average OCR for a healthy group in each state, and blue bars are for the OCR of the DEP group in each state. OCR features are extracted from dFNC of just pre-ECT (significant difference in state 2, corrected *p* = 0.015). **(B)** OCR features extracted from dFNC of just post-ECT (significant difference in state 2, corrected *p* = 0.03). In state 2, ECT had significantly changed the OCR value of HC and DEP before applying ECT (HC > DEP) compared with after applying ECT (HC < DEP). **(C)** It shows the traveled distance between the DEP and HC groups in pre-ECT and post-ECT. In pre-ECT, the traveled distance of the HC group is significantly higher than the DEP group (*p* = 0.04). After applying ECT, the HC group has higher traveled distance than the DEP group, but this difference is not significant. The significant difference that passes the multiple comparisons is marked by asterisks.

Figures 3A,B show the OCR values of HC and DEP groups in different states for pre-ECT and post-ECT, respectively. Only OCR of state 2, with relatively higher CCN/DMN functional connectivity, shows a significant difference between DEP and HC. We found pre-ECT DEP spent less time in state 2 relative to HC (FDR corrected *p* = 0.015), while the pattern reversed after ECT and patients with depression spent more time in state 2 (FDR corrected *p* = 0.03). Moreover, we compared the traveled distance between DEP and HC groups in pre-ECT and post-ECT (Figure 3C). The results showed that, in pre-ECT, the HC group traveled significantly more distance compared with the DEP group (*p* = 0.04). In post-ECT, again, the traveled distance of the HC group is higher than the DEP group, but this difference is not significant.

### The Link Between Pre-ECT OCR and the Effectiveness of ECT

**Figure 4:**
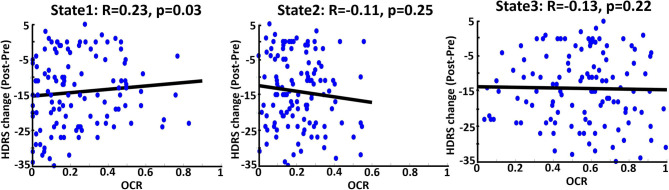
Correlation between OCR values from just pre-ECT and reported HDRS change (Pre-post) in three states. Blue dots are referred to 119 DEP individuals. The bold black line is the fitted curve. R indicates the fitted line slope in each state. As it is shown, state 1 (the state with the lowest CCN/DMN connectivity) significantly predicts the change of the HDRS based on OCR values; less OCR value corresponds to more change of HDRS.

To find associations to investigate whether applying ECT would be effective, we correlated the calculated OCR of 119 patients with their associated HDRS change (post_HDRS-pre_HDRS) by controlling the age, gender, a scanning site, and the number of treatments. As shown in Figure 4, which is the correlation of just pre-ECT OCR with HDRS, only the OCR of state 1 is the significant predictor (*R* = 0.22, FDR corrected *p* = 0.03). In more detail, we found those patients who spent more time in state 1, with relatively lower CCN/DMN functional connectivity, showed less reduction in their HDRS. We did not find a significant link between the pre-ECT traveled distance and the HDRS change.

## Discussion

This study used rs-fMRI of 119 subjects with a depressive episode and received ECT and 61 healthy subjects to assess longitudinal changes of brain dynamics associated with treatment response. We used dFNC features extracted from CCN and DMN and clustered them into three different states. Using a data-driven method of analyzing dFNC in CCN and DMN, we demonstrated that these brain networks are highly dynamic in both pre- and post-ECT states. This finding agrees with previous studies on MDD that have provided evidence of dynamism in CCN and DMN (Repple et al., 2020; Yang et al., 2020; Chen et al., 2021; Sendi et al., 2021a). Previous studies differentiated MDD from the HC group, focusing on within-DMN and within-CCN functional connectivity. For example, by focusing on sFNC information, one study reported an increase in within-DMN connectivity for MDD (Posner et al., 2016), while another study reported a decrease in this connectivity of the network (Li et al., 2020; Wang J. et al., 2020). Additionally, recent studies, using rs-fMRI, have suggested an association of depression with abnormal functional connectivity in CCN network (Schlösser et al., 2008; Vasic et al., 2009; Sheline et al., 2010; Veer et al., 2010; Alexopoulos et al., 2013; Clasen et al., 2014). Other studies focusing on the CCN network reported attenuated connectivity of that network in remitted MDDs (Stange et al., 2017; Jiao et al., 2020). Finally, a study tried to predict the antidepressant response in MDDs focusing on within-CCN and within-DMN networks and reported low- and high-resting functional connectivity within-CCN and within-DMN, respectively (Alexopoulos et al., 2012). While previous studies mainly focused on within-DMN and within-CCN functional connectivity, the current study might provide new evidence about the role of CCN/DMN connectivity in depression. Elaborating more on this, our results show that the state with significantly higher CCN/DMN connectivity plays an important role in discriminating between DEP and HC groups both in pre-ECT and post-ECT conditions.

We also investigated the effect of ECT on evaluating the temporal dynamic activity of the brain after implementing ECT in post-ECT conditions. We found that the OCR of DEP is significantly higher than the HC participants in this condition. This means that, after ECT, the patients with depression spent more time in state 2 than the HC group. Similar to pre-ECT state 2, post-ECT state 2 shows the highest CCN/DMN connectivity than other states. That might provide new insight into the effect of ECT on the CCN/DMN connectivity by regulating the temporal dynamics of these brain networks. Moreover, post-ECT state 2 has relatively higher within-CCN connectivity. This is in contrast to previous studies that reported reduced within-CCN connectivity associated with the antidepressant state in a relatively small dataset (*N* = 16) (Alexopoulos et al., 2012). While this inconsistent result could partially be due to the small number of patients, we assume that this inconsistent result is driven by the focusing of sFNC, estimated by averaging from the entire time series. In the current study, we showed a disrupted (i.e., both increase and decrease) pattern within CCN connectivity might provide an explanation for this inconsistent result and might provide a good reason for analyzing dFNC information (Sendi et al., 2021a).

Evaluating the effectiveness of ECT, i.e., identifying patients as potential remitters or non-remitters before implementing ECT, would be valuable from the clinical perspective (Van Waarde et al., 2015). On the one hand, many studies have correlated baseline clinical characteristics with an MDD status outcome (Perlman et al., 2019; Kennis et al., 2020). Such analyses are based on group-level analysis rather than individual patient-level aspects (Ozomaro et al., 2013). There is a need for new metrics, which are associated with ECT outcomes. On the other hand, selecting a feature among many MRI metrics is difficult because they focus on non-overlapping aspects of brain function (Leaver et al., 2018). Although some metrics are based on functional connectivity of fMRI data, they are focused on static brain region connections (Leaver et al., 2018). Therefore, the use of metrics based on dFNC and using the correlation analysis of such metrics with behavioral and clinical data could link with an ECT outcome. In this study, we were able to find associations between the effectiveness of ECT before applying it and HDRS scores. To this aim, we correlated the HDRS change with just pre-ECT OCR of DEPs and found that brain dynamics in state 1 is the predictor (Figure 4). We found a significant correlation between OCR and HDRS change with a positive slope, which means that the more OCR in pre-ECT state 1, with relatively less CCN/DMN, equals fewer HDRS changes. Therefore, DEPs who spent more time in pre-ECT state 1 are less likely to respond to ECT. Interestingly, the main characteristic of state 1 is that this state has the lowest connectivity between CCN/DMN relative to state 2 and state 3 (Figure 2A and Table 3). Also, the results of the effect of ECT showed that ECT had increased the amount of time that DEPs were spending in post-ECT state 2, with higher CCN/DMN connectivity. As such, we can conclude that the results of this finding are in line with the result of the effect of ECT, since spending time in the state with the minimum CCN/DMN correlation is not good, and ECT increased the amount of time DEPs spending in the state where the CCN/DMN correlation is maximum. Moreover, while previous studies focused on the sFNC to find a link between rs-fMRI data and an ECT outcome (Wei et al., 2018; Hill et al., 2020; Wang D. et al., 2020; Sinha et al., 2021), the current study is the first attempt, using dFNC information to predict the ECT outcome.

Finally, we extracted the total traveled distance metric from pre- and post-ECT dFNC, using Euclidean distance. This metric shows the dynamic of the brain since it measures the distance traveled (i.e., changes in FNC over time) between each subsequent dFNC window. In the pre-ECT condition, we found that DEP traveled distance is significantly lower than HC one. This means that DEP FNC changes less than HC FNC or DEP patient rs-fMRI is less dynamic than HC rs-fMRI. This is consistent with the previous finding that shows functional connectivity estimated from rs-fMRI of patients with MDD is less dynamic than the HC group (Kaiser et al., 2016). But this difference is not significant between DEP and HC after ECT. In other words, ECT decreases the traveled distance difference between HC and DEP. Therefore, we can conclude that ECT makes the brain activity of DEPs more dynamic.

### Limitations

There are a few limitations in the current study. First, we did not directly measure whether the participants were awake or closed their eyes during the scanning. Concerning this issue, we used the questionnaire and self-reports provided by subjects after the scanning finished. Based on the literature, this issue might affect our results (Agcaoglu et al., 2019). To address this, it is possible to extend the dynamic functional connectivity approach to assess when the eyes of the participants were closed, or they were exhibiting aspects of drowsiness (Allen et al., 2018). dFNC approaches have already shown promise in predicting measures of drowsiness (Damaraju et al., 2020). Moreover, our data were collected in two different sites, and the ECT protocol, including the number of treatments and the use of concurrent psychotropic medications, was different in these two sites. Addressing this issue, we tried to consider these differences by including sites as a covariate in our analysis to control their effect on the results. Despite the fact that HDRS is generally utilized in scaling the depression symptom severity, this score relies upon the skill and knowledge of the interview (Sharp, 2015). Since the data in this study come from two separate sites, each with its own set of raters, this may cause values of HDRS to vary and be inaccurate across sites.

## Conclusion

This study evaluated dynamic functional network connectivity of DMN and CCN, using rs-fMRI data of DEP patients experiencing ECT treatment. Focusing on CCN and DMN networks and clustering the brain dFNC to three different states, we found brain activity in these networks is highly dynamic. Comparing the OCR feature extracted from dFNC of these two networks between DEP and HC groups, we found that HC group prefers to spend more time in a state where the connectivity between CCN and DMN is the maximum. Moreover, we found that ECT causes an increase in the amount of time DEP patients spend in the state in which the CCN/DMN functional connectivity is maximum. In addition, we could significantly find associations with the effectiveness of the ECT, using just pre-ECT brain activity. We found that the more time the participants spend in the state in which the correlation of CCN/DMN is minimum, the less HDRS change they have, and the less effectiveness of ECT they would experience. Finally, we found that the distance that the DEP patients travel before ECT is significantly lower than the distance they travel after ECT compared with the HC group. While this difference was not significant after ECT, this suggests an increase in brain dynamics after implementing ECT. In brief, this study provides a focus on functional connectivity dynamics of CCN and DMN network of the DEP patients and introduces CCN/DMN connectivity as a biomarker, which is associated with the effectiveness of ECT.

## Data Availability Statement

The raw data supporting the conclusions of this article will be made available by the authors, without undue reservation.

## Ethics Statement

The studies involving human participants were reviewed and approved by University of New Mexico (UNM) and the University of California Los Angeles (UCLA). The patients/participants provided their written informed consent to participate in this study.

## Author Contributions

HD and MS developed the study, conducted data analysis, interpreted the results, and wrote the original manuscript draft. ZF and SQ pre-processed the data. RE and KN collected the data. CA collected the data and provided a critical review of the initial draft. SR, PR-P, LB, and HM provided a critical review of the initial draft. VC developed the study, interpreted the results, edited the original draft, and provided critical review to the initial draft. All authors approved the final manuscript.

## Conflict of Interest

The authors declare that the research was conducted in the absence of any commercial or financial relationships that could be construed as a potential conflict of interest.
